# Outcomes in very elderly ICU patients surgically treated for proximal femur fractures

**DOI:** 10.1038/s41598-024-51816-y

**Published:** 2024-01-16

**Authors:** Annika Heuer, Jakob Müller, André Strahl, Florian Fensky, Rikus Daniels, Pauline Theile, Karl-Heinz Frosch, Stefan Kluge, Jan Hubert, Darius Thiesen, Kevin Roedl

**Affiliations:** 1 Department of Trauma and Orthopedic Surgery, University Medical Center Hamburg-Eppendorf (UKE), Martinistraße 52, 20246 Hamburg, Germany; 2 Department of Intensive Care Medicine, University Medical Centre Hamburg-Eppendorf (UKE), Martinistraße 52, 20246 Hamburg, Germany; 3 Department of Anaesthesiology, Tabea Hospital, Hamburg, Germany

**Keywords:** Disability, Bone, Disease prevention, Fracture repair, Geriatrics, Prognosis, Quality of life

## Abstract

Proximal femur fractures (PFF) are a common injury in elderly patients that significantly impact mobility and daily living activities. Mortality rates in this population are also high, making effective treatment essential. Recent advances in intensive and geriatric care have enabled complex surgical interventions that were previously not feasible. However, there is a lack of studies focusing on outcome parameters in very elderly patients (≥ 90 years) who receive intensive care treatment following PFFs. In this retrospective study, we analyzed multi-layered data of 148 patients who were 90 years or older and received intensive care after trauma and orthopedic surgical treatment for PFFs or periprosthetic fractures between 2009 and 2019. All patients received a 365-day follow-up. To identify potential predictors of mortality, all deceased and surviving patients were subjected to multiple logistic regression analyses. We found that 22% of patients deceased during in-hospital care, and one-year survival was 44%. Independent predictors of one-year all-cause mortality included higher CCI and SOFA scores at ICU admission. Overall, 53% of patients who resided in private dwellings prior to admission were able to return home. Our study highlights the utility of using CCI and SOFA scores at ICU admission as prognostic indicators in critically ill very elderly patients who undergo surgical treatment for PFFs. These scores can provide valuable insight into the severity of illness and potential outcomes, which can inform resource allocation, prioritize endangered patients, and aid in end-of-life discussions and planning with patients and their families. Our findings can help improve the management of PFFs in very elderly patients and contribute to optimized patient care.

## Introduction

Proximal femur fractures (PFFs) are a common occurrence among elderly patients over the age of 75 and represent a detrimental life event that undermines their already compromised independence^1,2^. Following a PFF, only 40–60% of elderly patients regain their prior level of mobility and ability to perform activities of daily living^2^. The hospitalization, rehabilitation, and potential need for long-term care impose a significant socioeconomic burden on the healthcare system. Previous research has shown that one out of six patients living in their own home prior to the fracture must permanently relocate to a nursing home after experiencing a PFF^3,4^. PFFs can be categorized into fractures of the femoral head or neck and the pertrochanteric, intertrochanteric, or subtrochanteric region. The choice of surgical intervention, such as arthroplasty or internal or external fixation techniques like intramedullary nailing or sliding hip screws, will vary based on the affected region and individual patient factors and should ideally be performed within the first 24 h^1,5,6^. Geriatric patients with PFFs have a high risk of early death within the first 30 days, with mortality rates ranging from 7% to as high as 20–28% in the first year after injury^7,8^. The presence of multiple comorbidities and functional limitations only exacerbates the impact of the acute injury. Advances in intensive and geriatric care now allow for more complex surgical interventions in critically ill and geriatric patients, previously not feasible. Partly in a supportive function, intensive care treatment enables further surgical treatment in the first place^9^. Therefore, more critically ill and geriatric patients can undergo needed surgical interventions with subsequent submission for intensive care treatment^10^. Analysis of over 356,000 visits to the German emergency departments showed that the need of treatment, further hospitalization as well as admission to an intensive care unit increased significantly with age^11^. The study supports the increasingly louder demand to adapt treatment and processes to an aging patient population.

So far, no studies focused on outcome parameters of very elderly patients ≥ 90 years who received intensive care treatment following PFFs. Specifically, the one-year all-cause mortality rate in this patient population has not been studied. This study aims to investigate the clinical characteristics and outcomes, including in-hospital and one-year mortality, of critically ill nonagenarians in a large university hospital with a focus on tertiary care.

## Methods

### Study design and ethics

In this study, we retrospectively analysed data of patients who were 90 years or older and received trauma and orthopedic surgical treatment for a proximal femur or periprosthetic fracture between 2009 and 2019. Inclusion criteria for the cohort was post-operative admission to the tertiary Department of Intensive Care Medicine (ICU) of the University Medical Centre Hamburg-Eppendorf. The following data were extracted from the electronic patient data management system (Integrated Care Manager [ICM], Dräger Medical, Lübeck, Germany): Age, gender, type of residence before admission and after discharge, comorbidities, surgical details (diagnosis and intervention), ICU treatment (including laboratory results, vital parameters, treatment modalities and organ support, blood transfusions), discharge information, all-cause ICU- and in-hospital mortality.

The simplified acute physiology score II (SAPS II) was used to access severity of illness in all patients^12^. The SAPS II score which was developed in 1993, is a tool to predict all-cause hospital mortality of patients requiring ICU treatment and includes 12 physiological variables, age, type of admission, and three variables related to underlying disease. The worst value of the physiological variables during the first 24 h of ICU admission was used for calculation. The score ranges from 0 to 163, with 29, 40, and 64 points corresponding to an estimated in-hospital mortality risk of 10%, 25%, and 75%, respectively^12^.

Comorbidities were analysed using the Charlson Comorbidity Index (CCI)^13,14^. The CCI, which was developed in 1987, is used to predict ten-year all-cause mortality in patients with multiple comorbidities and includes 19 individual pathologies. Depending on their weight, individual pathologies are scored with 1–6 points and are interpreted as severe if ≥ 5 points are awarded. Patients with higher CCI scores were found to have a higher likelihood of death within the following 10 years (score “1” = 25%, “2” = 48% and “ > 3” = 59%)^13^. Recent studies also showed good fit to predict short term and in-hospital mortality of patients with PFF^14^. Using the Sequential Organ Failure Assessment (SOFA), degree of organ failure over time was rated^15,16^. The SOFA score, which was established in 1994, predicts ICU mortality using a five-point scale for six different independent items, including respiratory, cardiovascular, hepatic, renal, coagulation, and neurological systems^15^. The total score ranges from 0 (physiological function) to 24 (worst value), and each one-point increase corresponds to an approximately 1.35-fold increase in the risk of ICU death. Although the SOFA score was originally developed for patients with sepsis, recent data have shown that it has similar diagnostic accuracy for both surgical and non-surgical subjects^16^.

At admission and regularly during ICU treatment, laboratory tests, blood gas analysis, vital functions, ventilation parameters, pharmacological circulatory support, transfusions, and neurological status were recorded. Follow-up regarding all-cause mortality and living situation was obtained after discharge at 28-, 90-, and 365-day intervals.

The Ethics Committee of the Hamburg Chamber of Physicians (Ethikkomission der Ärztekammer Hamburg) approved the study (No.: 2021–300,116-WF). Due to the retrospective nature of the study and the deidentified study data, the need for explicit informed consent was waived by the ethics Committee of the Hamburg Chamber of Physicians (Ethikkomission der Ärztekammer Hamburg). The study was conducted in compliance with the Declaration of Helsinki as well as in accordance with local guidelines and regulations.

### Diagnosis and treatment

The study included proximal femur fractures, including femoral neck, pertrochanteric, and subtrochanteric fractures, as well as fractures in the vicinity of pre-existing hip replacements. The diagnosis was established using radiographic or computed tomographic imaging, which were reviewed by a board-certified radiologist and trauma/orthopedic surgeon.

**Figure 1:**
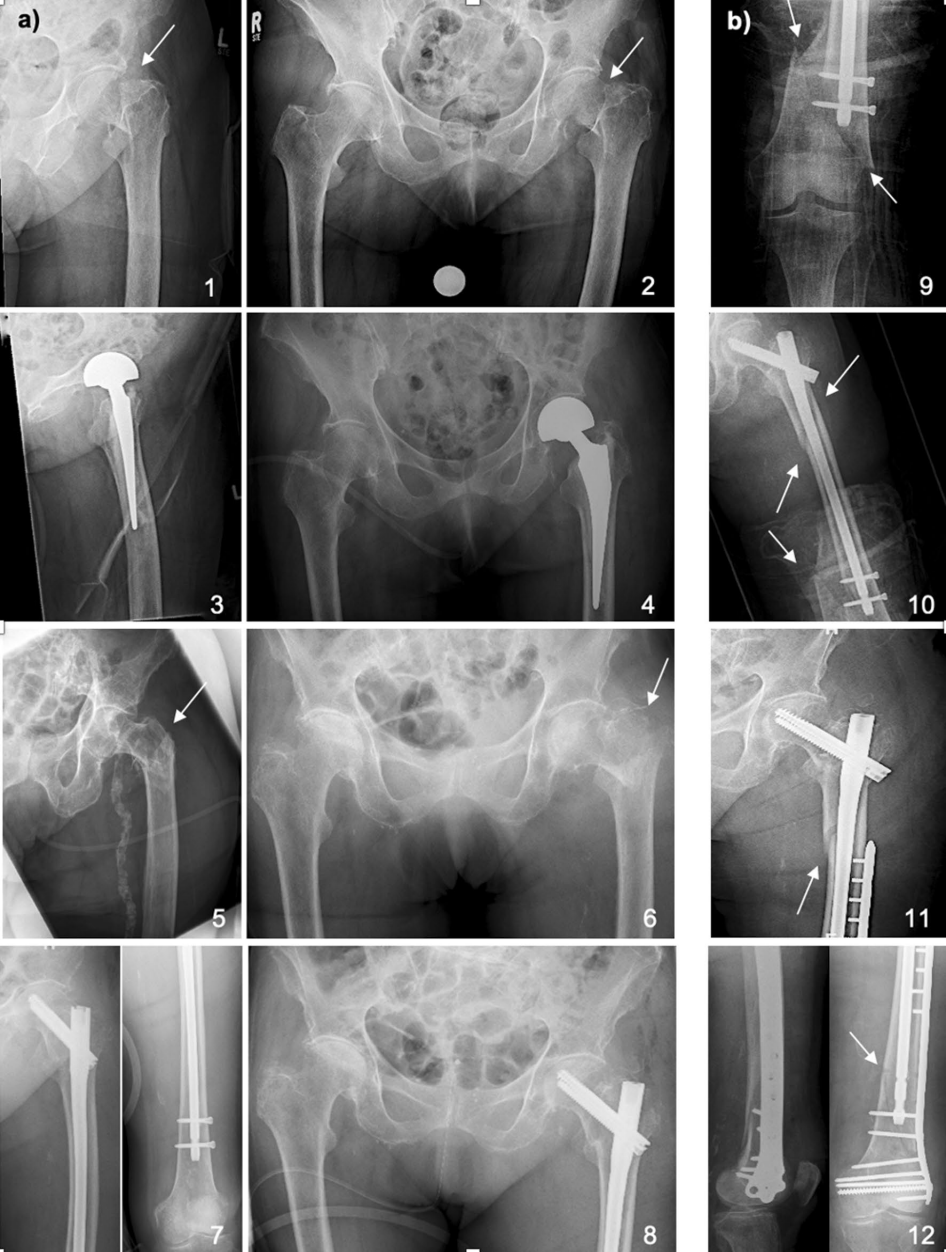
Operative intervention in patients presenting with (**a**) PFFs or (**b**) periprosthetic femur fracture. (**a**) First case^1–4^: A 94-year-old women was admitted to the ER with clinical signs of a PFF after falling at her private apartment. Radiographs of the left hip joint axial^1^ and the pelvis^2^ showed a medial femoral neck fracture (arrows). Due to already impaired walking ability and osteoporosis, a cemented hemi-arthroplasty was implanted. The patient recovered for 4 days at our intensive care unit (ICU). Radiographs 3 and 4 show postoperative results on day two. The patient was able to return home after specialized geriatric rehabilitation. Second case^5–8^: A 97-year-old women with dementia was administered to our ER after falling during transition at her assisted living facility. Radiographs of the left hip joint axial^5^ and the pelvis^6^ showed a multifragmentary pertrochanteric fracture (arrows). After closed reduction and internal fixation using a femur nail, the patient spent 24 h at the ICU. Postoperative radiographs 7 and 8 show results on day two. The patient was discharged into specialized geriatric rehabilitation. (**b**) At the rehabilitation facility the patient from case 2 refused weight bearing after a few days with initial walking ability and an unobserved fall was suspected. Radiographs of the pelvis and femur showed a distal supracondylar multifragmentary periprosthetic femur fracture with a rotary wedge reaching the subtrochanteric region (see arrows in image 9 and 10). Osteosynthesis to stabilize the distal fracture fragment was performed using a plate and postoperative recovery took place for two days at our ICU. Images 11 and 12 show postoperative results on day two. Due to prolonged recovery and increased need for care the patient was discharged to a short-term-care facility needing higher nursing intensity.

For patients with native joint fractures, treatment consisted of either osteosynthesis (nailing) for per- or subtrochanteric fractures, or hemi-hip-arthroplasty/total hip arthroplasty based on individual lifestyle and health factors for femoral neck fractures (Fig. 1a). In cases of periprosthetic fractures, osteosynthesis, aseptic one-stage implant revision surgery, or a combination of the two was conducted (Fig. 1b). The procedures were performed by board-certified trauma or arthroplasty specialists at our Level 1 trauma center.

Patients were preoperatively evaluated by a board-certified anaesthesiologist for operative risk factors and health status and scheduled for postoperative critical care if necessary. Whilst no strict protocol is in place for admitting nonagenarians and centenarians to the ICU after surgical treatment for PFF, we take a multidisciplinary approach specific to each patient. Patient will, comorbidities, health status as well as intra- and postoperative parameters (hemodynamic and pulmonary changes) are discussed within the multidisciplinary team of anesthesiologist, intensivist, and treating surgical team before transferring a patient to the ICU or surgical ward after initial post-anesthesia care unit (PACU) monitoring.

In accordance with national treatment guidelines, surgical intervention was performed within 24 h, unless there were contraindications such as anticoagulants or unfavourable health status^17^. Coagulation monitoring and optimization, as well as necessary treatment delays, were planned in collaboration with a specialized haemostasis therapy team. The utilization of conservative treatment is a rare exception in cases where a patient's preference prohibit surgery or a detrimental combination of comorbidities, such as severe cardiovascular disease, pneumonia, ASA 4 classification, malnutrition, or high frailty index, strongly indicate a low likelihood of surviving the peri-operative phase^17^.

Postoperatively, patients received treatment at one of our 12 specialized ICUs with a total capacity of 142 beds and a high level of care. After sufficient recovery, patients were transferred to our surgical wards. All patients received a daily session of physiotherapy and were transferred preferably to rehabilitation, alternatively to specialized geriatric care facilities for further recovery or their care facilities or homes.

### Statistical analysis

All analyses were performed using SPSS version 29 (IBM, Armonk, New York, NY, USA) for Windows. Continuous variables are expressed as mean ± standard deviation (SD), while categorical variables are expressed as a number and percentage (%). Normality distribution of data was tested using the Shapiro–Wilk test. To compare patients in terms of continuous variables, the Student’s *t*-test for independent samples was used for normally distributed data. The Mann − Whitney U test was used with non-normally distributed data, and chi-squared or Fisher’s exact tests were applied for categorial variables. The probability of 365 days survival was estimated as a function of time using the Kaplan–Meier survival method with a 95% confidence interval. To identify potential predictors of mortality, all deceased and surviving patients were subjected to multiple logistic regression analyses. To control prerequisites of the regression, Pearson correlation for continuous and Spearman correlation for nonparametric variables were applied. Correlations among predictors should amount less than 0.7. Both, continuous and categorical variables were offered to the logistic regression model. Predictors were logarithmised (Log base 10) for analysis if they were not normally distributed. The analyses used the 'stepwise backwards logistic regression' method applying the maximum likelihood function to compensate for nonlinearity regarding the influence of continuous variables. The goodness of fit was judged with Nagelkerke’s R^2^. In accordance with accepted standards, statistical significance was set to a 2-tailed *p* value of < 0.05.

## Results

### Demographic characteristics and surgical treatment

During the study period from 2009 – 2019 we could identify 1108 patients ≥ 90 years admitted to the ICU. Of those 13.3% (n = 148) were admitted for trauma and orthopaedic surgical treatment and had proximal femur or periprosthetic fracture. Whitin the timeframe, a total of 246 nonagenarians and centenarians were surgically treated due to a PFF. The rate of postoperative ICU admission was 51% between 2009–2014 and 70% between 2015 – 2019, whilst number treated, and age were 127 versus 119, and 95 versus 94 years for the respective time spans. Patients not admitted to the ICU were moved to the surgical ward after postoperative monitoring at the post-anesthesia care unit (PACU). A total of 148 patients were eligible for retrospective analysis.

The mean age at admission was 94.2 ± 3 years [90 – 106 years], and the majority of patients were female (81%, n = 117). Preoperatively, 41% of patients (n = 61) were residing in a private dwelling, 46% (n = 68) in a long-term care facility, and 14 patients (10%) were living in an assisted living facility. CCI in the study cohort ranged from 0 to 7 points, with an average of 1.5 ± 1.5. 76% (n = 112) of patients had a preoperative ASA score of 3 ± 0.5. Table 1 provides additional demographic and disease characteristics of the patient population.

**Table 1:** Shown are baseline characteristics of the study population.

Variables	All (n = 145)	365 d Survival (n = 64)	Deceased before 365 d (n = 81)	*p* value
Age (years) (mean ± SD)	94.1 ± 3.0	93.9 ± 3.1	94.3 ± 3.0	0.38
Female (n, %)	117, 80.7%	53, 82.8%	64, 79.0%	0.67
BMI (kg/m^2^) (mean ± SD)	22.82 ± 3.87	22.80 ± 3.97	22.84 ± 3.81	0.95
Comorbidities				
CCI (points)	1.5 ± 1.5 (range, 0–7)	0.9 ± 1.0	1.9 ± 1.6	** < 0.001**
Arterial hypertension (n, %)	106, 73.1%	46, 71.9%	60, 74.1%	0.85
Chronic kidney disease (n, %)	36, 24.8%	10, 15.6%	26, 32.1%	**0.032**
Aortic valvular stenosis (n, %)	9, 6.2%	5, 7.8%	4, 4.9%	0.5
Atrial fibrillation (n, %)	56, 38.6%	24, 37.5%	32, 39.5%	0.86
Congestive heart failure (n, %)	35, 24.1%	10, 15.6%	25, 30.9%	**0.05**
Diabetes mellitus (n, %)	12, 8.3%	5, 7.8%	7, 8.6%	1.0
Lung disease (n, %)	8, 5.5%	1, 1.6%	7, 8.6%	0.07
COPD	5, 3.4%	0, 0.0%	5, 6.2%	0.06
Dementia (n, %)	48, 33.1%	14, 21.9%	34, 42.0%	**0.013**
PAD (n, %)	8, 5.5%	2, 3.1%	6, 7.4%	0.46
Prevalent cancer (n, %)	8, 5.5%	1, 1.6%	7, 8.6%	0.07

*d* days, *CCI* Charlson comorbidity index, *COPD* chronic respiratory pulmonary obstruction, *PAD* peripheral artery disease.

Bold print indicating significant variations between the two groups.

The most common type of fracture was at the femoral neck (53%, n = 78), followed by fractures of the pertrochanteric or intertrochanteric region (30%, n = 44), periprosthetic fractures (14%, n = 20), and subtrochanteric fractures (3%, n = 5). 52% of patients (n = 77) underwent surgery outside of regular hours (7 p.m. – 8 a.m.) or on weekends. The median surgery time was 80 ± 40 min, and no correlation was found between surgery time of day or duration and mortality.

### ICU treatment characteristics

All patients received postoperative intensive care treatment either due to pre-existing critical illness or an increased risk of perioperative adverse events identified during surgery. On average, patients spent 12 ± 7 days hospitalized and 2.4 ± 3 days in the ICU. At admission to the ICU, the SAPS II scores of the patient cohort ranged from 18 to 67 points (mean of 37.6 ± 11.6), and the SOFA scores ranged from 0 – 12 points (mean of 2.9 ± 3). The CCI ranged from 0—7 (mean 1.5 ± 1.5). Sixteen patients (11%) required invasive ventilation and seven (5%) non-invasive ventilation, with an average duration of less than 24 h.

At admission, the average venous lactate was 1.5 ± 1.2 mg/dl, haemoglobin was 10 ± 1.4 g/L and the INR was 1.1 ± 0.15. Three patients (2%) required postoperative transfusions of red blood cells due to bleeding, but no additional surgical interventions were necessary. On average, 42% of ICU patients (n = 62) received 1.7 ± 0.86 red blood cell transfusions. Seven patients (5%) received transfusions of three or more units. There was no correlation between haemoglobin levels or transfusion rates and mortality. Additional patient information, including laboratory results and treatment details, can be found in Table 2.

**Table 2:** Shown are laboratory and blood gas analysis results comparing value at admission and last measurement before discharge or patient death.

Variables	All	Patients with 365-day survival	Patients deceased before 365 days	*p* value
Vital functions – at admission				
Glasgow coma scale	13.4 ± 3.9	14.0 ± 3.2	12.9 ± 4.2	0.07
Body temperature (°C)	35.9 ± 1.0	36.0 ± 0.9	35.8 ± 1.0	0.23
Heart rate (beats/minute)	87.3 ± 25.2	82.1 ± 18.6	91.3 ± 28.8	**0.022**
Mean arterial pressure (mmHg)	86.3 ± 20.4	90.1 ± 19.1	83.3 ± 21.0	**0.045**
Vasopressor use				
Catecholamine	53, 36.6%	18, 28.1%	35, 43.2%	0.08
Epinephrine	3, 2.1%	0, 0.0%	3, 3.7%	0.25
Norepinephrine	53, 36.6%	18, 28.1%	35, 43.2%	0.08
Respiratory support – admission				
paO2/FIO2	473.0 ± 328.0	515.3 ± 327.1	437.6 ± 327.2	0.21
Invasive mechanical ventilation	16, 11.0%	4, 6.3%	12, 14.8%	0.11
Non-Invasive ventilation/HFNC	7, 4.8%	4, 6.3%	3, 3.7%	0.70
ICU Outcome				
Duration of ICU stay (days)	2.4 ± 3.3	2.14 ± 3.67	2.57 ± 2.94	0.43
Deceased at ICU	14 (9.7%)	–	–	–
Laboratory results				
Hemoglobin (g/dl) – admission	9.9 ± 1.3	9.9 ± 1.4	9.9 ± 1.3	0.94
Hemoglobin (g/dl) – last	8.7 ± 1.1	8.5 ± 1.0	8.8 ± 1.1	0.26
Leukocytes (Mrd/l)—admission	13.3 ± 5.9	12.8 ± 4.5	13.7 ± 6.8	0.35
Leukocytes (Mrd/l)—last	9.8 ± 4.5	9.0 ± 4.0	10.4 ± 4.9	0.08
Thrombocytes (Mrd/l)—admission	202.1 ± 100.9	199.2 ± 71.1	204.5 ± 120.3	0.75
Thrombocytes (Mrd/l)—last	174.9 ± 77.6	174.4 ± 63.4	175.4 ± 88.7	0.94
Creatinine (mg/dl)- admission	1.2 ± 0.6	1.1 ± 0.6	1.3 ± 0.7	**0.025**
Creatinine (mg/dl)—last	1.5 ± 1.4	1.3 ± 1.3	1.7 ± 1.5	0.09
CRP (mg/l) – admission	58.9 ± 38.6	57.8 ± 40.3	56.0 ± 37.2	0.78
CRP (mg/l) – last	107.2 ± 51.0	100.8 ± 48.7	112.7 ± 52.7	0.18
INR – admission	1.1 ± 0.2	1.1 ± 0.1	1.1 ± 0.2	0.72
INR – last	1.1 ± 0.2	1.1 ± 0.9	1.1 ± 0.2	0.12
Blood gas analysis				
paO2 (mmHg) – admission	101.6 ± 41.9	100.5 ± 41.8	102.5 ± 42.3	0.80
paO2 (mmHg) – last	87.0 ± 25.5	81.9 ± 21.9	91.6 ± 27.7	**0.05**
pvO2 (mmHg) – admission	37.8 ± 20.0	38.2 ± 19.4	37.5 ± 21.1	0.90
pvO2 (mmHg) – last	349.9 ± 202.6	377.6 ± 209.3	330.2 ± 203.1	0.58
pCO2 (mmHg) – admission	42.3 ± 42.3	40.8 ± 6.2	43.5 ± 8.9	**0.038**
pCO2 (mmHg) – last	39.5 ± 6.1	38.9 ± 4.9	39.9 ± 6.9	0.35
pH – admission	7.4 ± 0.1	7.4 ± 0.1	7.3 ± 0.1	0.08
pH – last	7,4 ± 0.1	7.4 ± 0.1	7.4 ± 0.1	**0.006**
Lactate (mmol/l) – admission	1.5 ± 1.2	1.2 ± 0.7	1.8 ± 1.5	**0.003**
Lactate (mmol/l) – last	1.2 ± 1.2	0.8 ± 0.4	1.5 ± 0.2	**0.001**

*CRP* C-reactive protein, *INR* international normalized ration, *pa* partial pressure in an arterial measurement, *pv* partial pressure in a venous measurement, *O*_*2*_ oxygen, *CO* carbon dioxide, *pH* pH value.

First and last known measurements before death or referral are included.

Bold print indicating significant variations between the two groups.

### In-hospital mortality

Fourteen patients (10%) deceased whilst postoperatively treated at the ICU. Patients of the deceased cohort are grouped as 'mortality group' (MG), surviving patients as 'survival group' (SG) for further comparison. Whitin the ICU MG, need of ventilative support was significant higher (50%) as for the ICU SG (17%, *p* < 0.005). After ICU transfer to surgical wards, eighteen more patients (12%) deceased during hospitalization. Overall, 32 patients (22%) deceased during in-hospital care.

The in-hospital SG showed significant differences compared with the in-hospital MG regarding SAPS II (SG: 35 ± 10; MG: 47 ± 13; *p* < 0.001), SOFA (SG: 2.3 ± 2.5; MG: 5.1 ± 3.3; *p* < 0.001) as well as lactate at ICU admission (SG: 1.3 ± 0.8; MG: 2.3 ± 1.9; *p* < 0.005) and CCI (SG: 1.3 ± 1.4; MG: 2.1 ± 1.6; *p* < 0.005). Within the in-hospital MG 60% of patients (n = 19) relied on ventilative support at ICU admission whereas only 14% of the in-hospital SG (*p* < 0.005).

Overall, 78% of patients (n = 115) survived surgical treatment and in-hospital medical care. We transferred 55% of patients (n = 63) to receive specialized geriatric rehabilitation. 53% of patients (34 of 64) who were previously self-supporting at a private living space could return home within the postoperative year.

#### Short-term follow-up

During the 28- and 90-day follow-up, 6 and 18 additional patients of those 115 patients who survived the in-hospital phase passed away. Both, the 28- and 90-day MGs showed prolonged ventilative support during their intensive care period (*p* < 0.05) compared to surviving patients. The 28-day SG showed significant differences compared to the 28-day MG in terms of SAPS II (SG: 35 ± 9.6; MG: 45 ± 13; *p* < 0.001) and SOFA score (SG: 2.2 ± 2.5; MG: 4.7 ± 3.5; *p* < 0.001) as well as lactate levels at ICU admission (SG: 1.2 ± 1; MG: 1.8 ± 2; *p* < 0.003) and CCI (SG: 1.3 ± 1.4; MG: 2 ± 1.5; *p* = 0.013). In a multiple binary logistic regression analysis, only SAPS II showed an independent positive predictive value for 28-day mortality (*p* = 0.002, R^2^ = 0.384, OR 1.085 [95%CI 1.031 – 1.142]).

### All-cause one-year mortality

**Figure 2:**
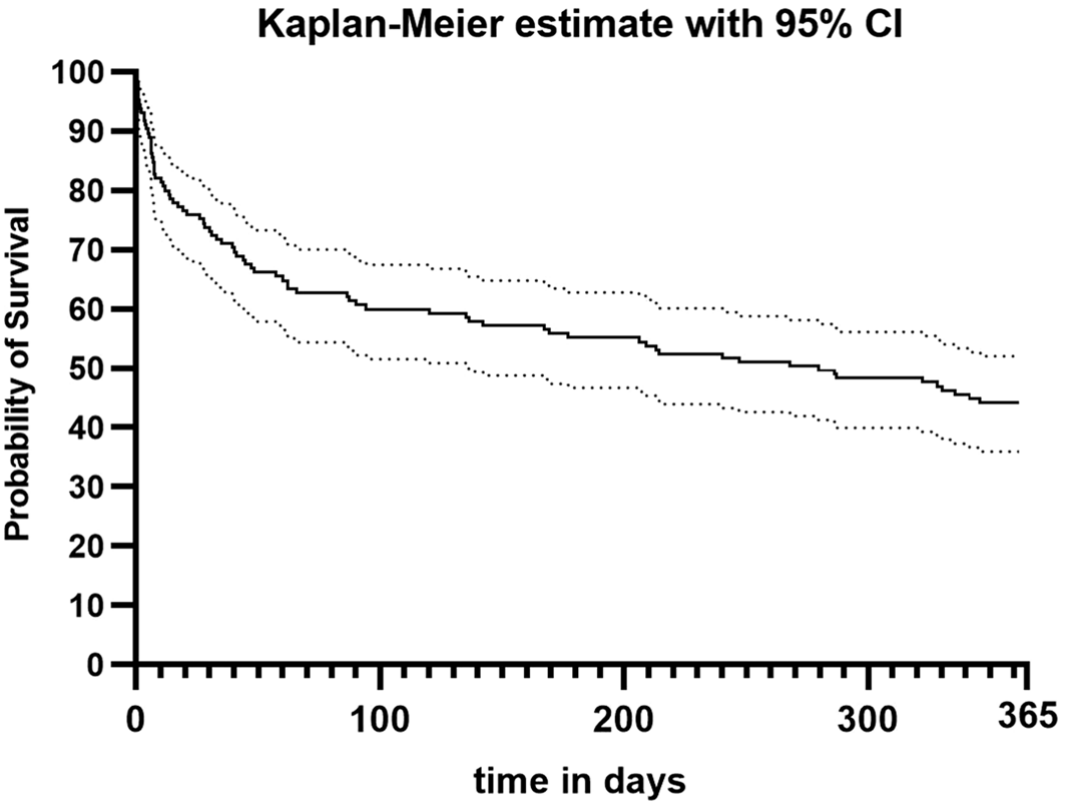
Shown is the one-year survival analysis as Kaplan–Meier plot. Probability of Survival in %; time in days.

One year survival of our cohort was 44% (n = 65) as seen in Fig. 2.

At ICU admission, patients belonging to the one-year SG showed a lower maximal heart frequency of 82 ± 19 beats per minute (bpm) than the MG with 91 ± 29 bpm (*p* < 0.05) as well as higher mean arterial pressure: SG 90 ± 19 mmHg and MG 83 ± 21 mmHg (*p* < 0.05) as seen in Table 2. The SG also showed differences compared with the MG regarding SAPS II (SG: 34.6 ± 9; MG: 40 SD ± 13; *p* < 0.003), SOFA score after 24 h (SG: 2.0 ± 2; MG: 3.5 ± 3; *p* < 0.002 but not respiratory support as seen in Table 3. Lactate level at ICU admission (SG: 1.1 ± 0.7; MG: 1.7 ± 1.5; *p* < 0.005) and CCI (SG: 0.9 ± 1; MG: 1.9 ± 2; *p* < 0.001) also showed significant differences. An advance directive was available in 17% of patients (n = 25) and had no influence on one-year survival rates.

**Table 3:** ICU Characteristics of patients with proximal femur fracture. Shown are values regarding disease severity and respiratory support.

Variables	All	365d Survival	Deceased before d 365 d	*p* value
Disease severity				
SAPS II – admission (pts.)	37.7 ± 11.6	34.6 ± 9.2	40.1 ± 12.6	**0.003**
SOFA – admission (pts.)	2.9 ± 3.0	2.1 ± 2.5	3.5 ± 3.2	**0.004**
SOFA – 24h (pts.)	2.9 ± 3.2	2.0 ± 2.2	3.6 ± 3.7	**0.002**
Respiratory support				
Invasive MV	24, 16.6%	8, 12.5%	16, 19,8%	0.269
Duration of MV (days)	0.71 ± 0.82	0.47 ± 0.60	0.83 ± 0.90	0.312

Significant are in value [bold].

*SAPS II* simplified acute physiology score, *SOFA* sequential organ failure assessment score, *pts* points, *MV* mechanical ventilation.

Data are expressed as n (%).

Table 3 depicts further values regarding disease severity and respiratory support.

The binary logistic regression model to investigate predictors of 365-day mortality was statistically significant (χ^2^ (4) = 28.12, *p* < 0.001, R^2^ = 0.248) identified only CCI (*p* = 0.004) and SOFA score (*p* = 0.037) as independent predictors with positive predictive values for all-cause one-year mortality as seen in Table 4. Performance metrics show a good statistical fit with AUC 0.783.

**Table 4:** Logistic regression for independent predictors of mortality in patients with PFF. Regression with “stepwise backwards” method.

Logistic regression	Predictor	*p* value	β (SE)	OR [95% CI]
Final model	SOFA	0.037	0.15 (0.072)	1.162 [1.009 – 1.338]
	Lactate	0.070	1.60 (0.883)	4.960 [0.878 – 28.014]
	Charlson comorbidity index	0.004	2.50 (0.880)	12.181 [2.172 – 68.324]
	Dementia	0.231	− 0.53 (0.439)	0.591 [0.25 – 1.398]
	Overall model evaluation Omnibus test	< 0.001		
	Goodness-of-fit test Hosmer–Lemeshow test	0.480		

*OR* odds ratio; *CI* confidence interval; *SOFA* Sepsis-related organ failure assessment score.

*Charlson Comorbidity Index, Lactate at admission were transformed prior to logistic regression analysis (Log base 10—logarithm).

## Discussion

**Figure 3:**
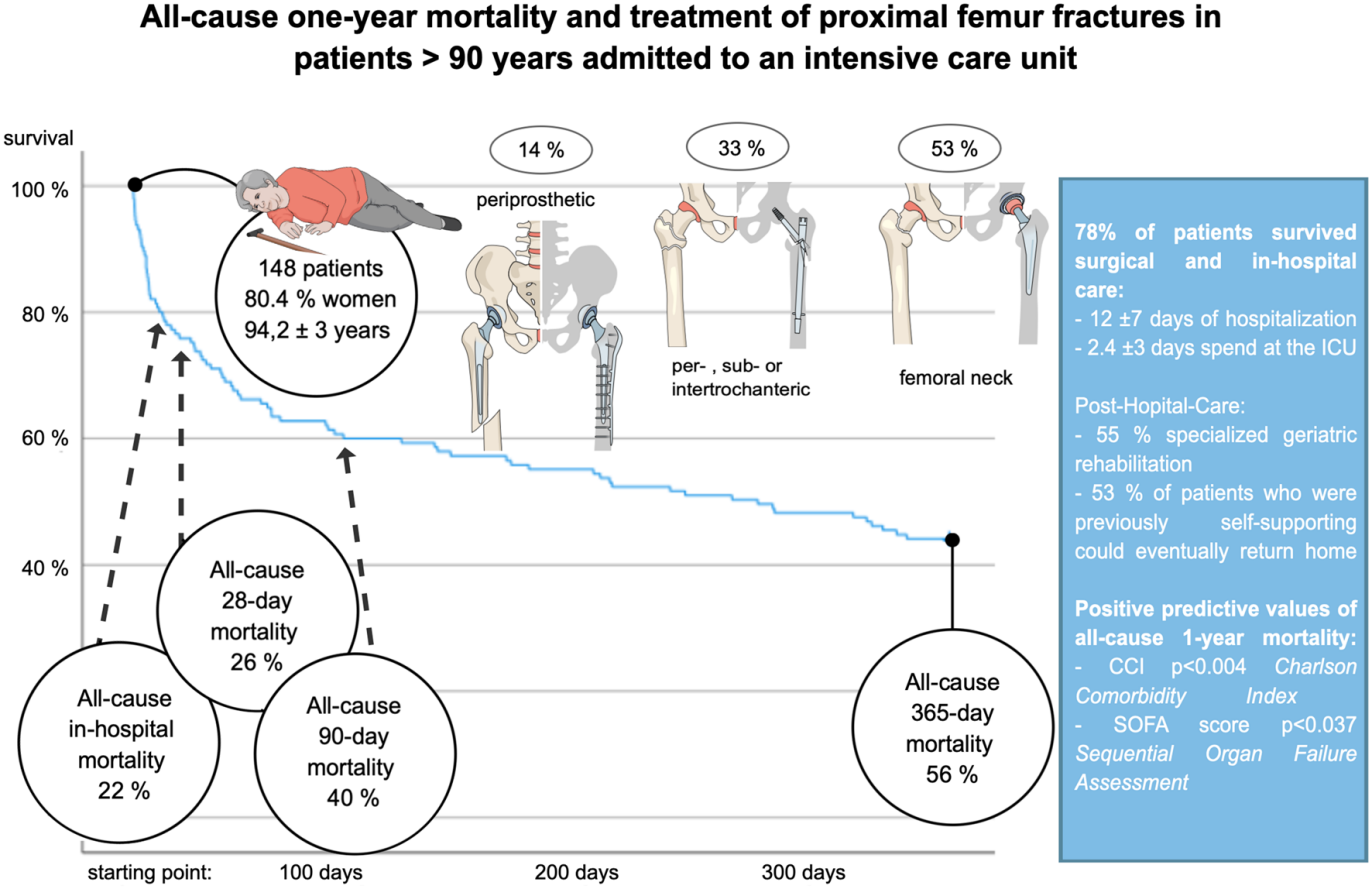
Shown is a concise overview of important study characteristics and results supporting further interpretation.

In this large study of 148 very elderly patients ≥ 90 years who underwent surgically treatment for PFFs or periprosthetic femur fractures and were consecutively admitted to the ICU, our main findings were: (A) 78% of patients survived surgical procedure and hospitalization, including ICU treatment; (B) 53% of patients who resided in private dwellings prior to admission were able to return home; and (C) Independent predictors of one-year all-cause mortality included higher CCI and SOFA scores at ICU admission (Fig. 3).

To our knowledge this is the first comprehensive study focusing on a very vulnerable sub-cohort of very elderly patients with PFFs treated in the intensive care unit (ICU). We found an all-cause in-hospital mortality of 22% and one-year mortality of 56% in the present study. Of interest is, this is twice as high as reported in contemporary European studies of elderly patients with PFFs: In a prospective multicenter study in Spain (997 cases, 2014 – 2016, mean age 84 years) in-hospital and 60-day mortality was reported to be 2 and 11% respectively^18^. Similarly, in a large German registry study (9780 patients, 2017 – 2019, mean age 84 years) the mortality rate was five percent in-hospital and 12% during their overall 120-day observation period^3^. International comparison shows a two percent in-hospital mortality in Taiwan (841 cases, 2016 – 2020, mean age 86 years)^19^ and in Turkey (335 patients, 2017 – 2019, mean age 83 years), short term (30 and 90 days) as well as one-year mortality were ten, twenty-two and 34% respectively^20^. Turgut et. al identified age > 90 years as independent prognostic risk factor for short and long-term mortality (*p* = 0.003)^20^. Focusing on contemporary cohorts composed of nonagenarians, a sub-analysis of the prospective study by Barceló et. al in Spain found in-hospital mortality of thirteen percent and cumulative mortality rates at 30-days, 3-months and 1-year of 20, 31 and 51% respectively (200 cases, 2009—2015, mean age 97 years)^18,21^. In a Swedish registry 30-day and 1-year mortality was found to be ten and seventeen percent (101, 2008 – 2012, 92 years)^22^. Internationally, in China two independent studies reported 6-month, 1- and 2-year mortality rates between 7 – 20%, 14 – 30% and 29 – 45% (184/144, 1997 – 2010/2014 – 2018)^23,24^.

The initially higher mortality rate in our study can be attributed to the specific cohort and institutional factors. First, our study only included patients treated in the ICU, leading to a selection of predominantly critically ill nonagenarians with a higher susceptibility to rapid health deterioration^25^. This is supported by Turgut et al., who found a significant increase in 30-, 90- and 365-day mortality for both admission to and length of ICU stay in their cohort (ICU admission rate of 55%) through univariate analysis^20^. Second, our level one trauma and orthopedic department is one of the largest tertiary referral centers in the area, resulting in the transfer of complex cases into our care, but also more frequent admission of critically ill patients to our emergency unit^26^. Additionally, our university medical center commonly treats patients with severe co-morbidities, including fall-related PFFs due to oncologic health issues, which implies the presence of serious underlying health issues. In conclusion, our study cohort is reflective of a typical contemporary PFF cohort of nonagenarians at a tertiary trauma and orthopedic center.

As life expectancy increases worldwide, a growing number of individuals reaching the mile stone of 90 years, are expected to live for several more years. According to the German federal statistical office (DESTATIS) and the human mortality database report, in 2020, the average mortality rate for German individuals aged 94 (which aligns with the median age of the sample cohort) was reported to be 27%^27,28^. In contemporary cohorts of nonagenarians, all-cause one-year mortality rates were found to be 20% (Italy, n = 433, median age of 92), 19% (Spain, n = 186, mean age of 93), and 26% (Danish cohort of 1905, n = 579, at time of analysis 93 years)^29–31^. Comparatively, admission with a PFF and consecutive ITS admission increases the risk of mortality twofold when compared to the general population, emphasizing the need for further understanding of specific risk factors in this vulnerable patient group. Calculations for the birth cohort of 2020 predict a mortality rate of 10 – 17% for men at age 94, which translates to a life expectancy of 4 – 5 more years, and an 8 – 14% mortality rate for women, which translates to life expectancy of 5 – 6 more years^27,28^. An aging population seeking disability-free years challenges the existing healthcare systems. Preparation to allow efficient and optimal geriatric care will be crucial in reducing morbidity and improving the quality of life for older individuals in the future.

Several comorbidities and patient characteristics have previously been linked to increased mortality after PFFs, including male gender, dementia, cardiac disease, and renal dysfunction^26,32,33^. However, our study found no significant association between mortality and male gender, COPD, or chronic lung disease. Subsequent multivariate logistic regression analysis of initially significant values showed that creatinine, lactate, vasopressor dependency, paCO2, signs of chronic kidney failure, heart rate, and mean arterial pressure at ICU admission did not independently predict one-year mortality. The relationship between comorbidities, laboratory results, and mortality remains controversial, with some studies finding significance but lacking further multivariable regression analysis^20,22,34^. Our study found no increased mortality with out-of-hours surgery compared to previous reports^35^.

The increase in ICU admissions in recent time seems to be attributed to the heightened concern surrounding unplanned ICU admissions after transferring patients to the surgical ward, a circumstance associated with elevated postoperative morbidity and mortality rates^36,37^. More than half of the cohorts’ patients received treatment during nonstandard hours, which is linked to reduced staff availability on general wards^38^. Acknowledging the significance of postoperative care, the Association of Anaesthetists of Great Britain and Ireland recommends a 1:4 nurse-to-patient ratio for optimal care when transferred to surgical wards^39^. Given the vulnerability of this elderly patient cohort and consequently high environment requirements, we suspect that the rising ratio of ICU-treated patients results from a more cautious approach, coupled with an increased availability of intermediate and high care capacity, rather than specific patient criteria.

While 78% of patients survive initial in-hospital treatment, nonagenarians face a higher mortality rate during follow-up with only 44% survival. An Italian cohort study of hospitalized nonagenarian patients (n = 124, median age of 93) showed a similar probability of being alive at one year (45%)^40^. Over 50% of patients aged 85 and older are considered frail, making them more susceptible to stressors due to limited resources^25^. Geriatric specialists support our hospital team to assess and monitor nonagenarian patients to minimize adverse events. Despite this, PFFs result in a permanent reduction in quality of life, with a high proportion of patients moving to nursing homes and losing their independence^3,8^. Studies reported that approximately one out of six patients who lived in a home-dwelling location before the trauma had to be permanently relocated into a nursing facility^3,41^. In our cohort, half of the patients who previously lived alone were unable to return home, causing a financial and psychological burden on patients and their families. Our ICU setting offers immediate and early physiotherapy which is continued on surgical wards to mobilize patients. This treatment strategy of early and daily rehabilitation was associated with higher discharge rates to private homes in a study in Finland^42^.

Assessing patient risk after admission for PFFs is crucial in predicting adverse events and complications and guiding patients and their families. This is especially important in critical illness cases requiring ICU treatment. PFFs and their aftermath can cause significant psychological stress, particularly when family members are required to make decisions in cases of rapidly declining health or dementia. Identifying factors that support informed decision-making and accurate assessment of the situation is essential in guiding patients, families, and determining appropriate treatment and care. Of interest is, that patients had only a low number of advance directives which is in line with previous studies in the intensive care setting^43^. However, in general early discussion of the patient's concrete wishes should be encouraged to ensure that treatment aligns with the patient's values, beliefs, and preferences.

- The SOFA score demonstrated independent predictive value for one-year mortality (*p* 0.037) in our cohort, with a range of 0 – 12 and mean value of 3.5 ± 3.2 for the MG and 2.1 ± 2.5 for the SG. Although initially designed for patients with sepsis, recent data suggests similar accuracy in both surgical and non-surgical subjects^16^. Clearly, ICU mortality is strongly associated with organ failure rate and severity which is tied to the SOFA score. A total SOFA score ≤ 4 indicates a high likelihood of discharge without adverse events, while a score ≥ 10 is associated with increased mortality. However, the range between these thresholds provides less precise discrimination, limiting the use of the SOFA score as a prognostic marker. A total SOFA score ≤ 4 indicates a high likelihood of discharge without adverse events, while a score ≥ 10 is associated with increased mortality. However, the range between these thresholds provides less precise discrimination, limiting the use of the SOFA score as a prognostic marker^44^. In a nutshell, it is clear that organ failure is associated with mortality, however, the lack of adequate discrimination at intermediate SOFA score values makes it an unreliable predictor of mortality^44^. The interpretation of the SOFA score should be based on its intended use, whether it is as a diagnostic tool, prognostic marker, or resource allocation aid. While it can provide valuable insight into patient severity and potential outcome, it should not be solely relied upon for prognostication. It can also aid in patient triaging and facilitate end-of-life discussions with families^44^.
- CCI has been found to have a significant predictive value for mortality after one year (*p* 0.004). In the study cohort, the mean value was 1.9 ± 1.6 in the one-year MG and 0.09 ± 1 for the SG. With an aging population, hospitals are facing an increase of patients with PFFs and multiple comorbidities, but ICU treatment also allows more critically ill patients to receive surgical care. Recent studies have shown that CCI can accurately predict short-term and in-hospital mortality in PFF patients^14,45^. Comorbidities in patients with hip fracture have been shown to increased 30-day postoperative mortality^46^. Schrøder et. al highlighted a comorbidity-related disparity of quality of in-hospital care which unintendedly led to an impaired patient prognosis. Such patients were less likely to receive the totality of recommended care, and rehabilitation with early preoperative optimization and mobilization impacted most^47^. The level of care dependency was found to be a determinant of quality of life and also of survival rates among comorbid patients with COPD, chronic heart failure or chronic renal failure^48^. The impact of comorbidities on receiving total recommended care is not limited to patients with PFF, and should be used to highlight the need for tailored clinical initiatives to ensure optimal patient care and best chances of survival^47^.

This study presents both strengths and limitations. Although the sample size is limited, it is the first extensive examination of the incidence of mortality among critically ill nonagenarians with PFFs. The single-center design restricts the generalizability to other healthcare settings, such as non-tertiary level hospitals. We acknowledge the disparities observed across nations which are shaped by diverse healthcare infrastructures, economic capacities, and cultural contexts and are often intertwined with the availability and allocation of resources. These differences underscore the importance of considering the resource landscape when interpreting and comparing outcomes on a global scale. To increase significance, it is recommended to validate the findings using a separate cohort in future studies. Additionally, the retrospective nature of the study and its focus on the ICU perspective restricts the available data on preoperative functional values, and there may be other influential factors impacting mortality that were not considered in this study. Also, only restricted demographic data regarding non-ICU admitted and conservatively treated patiens were available for analysis.

## Conclusions and implications

Our study demonstrates the utility of using CCI and SOFA scores at ICU admission as prognostic indicators in a cohort of 148 critically ill very elderly patients who underwent surgical treatment for PFFs. These scores can provide insight into the severity of illness and potential outcomes, which can inform resource allocation, prioritize endangered patients, and aid in end-of-life discussions and planning with patients and their families.

## Data Availability

The datasets used and/or analyzed during the current study are available from the corresponding author on reasonable request.

## Abbreviations

MG: Mortality group
SG: Survival group
CCI: Charlson comorbidity index
SOFA: Sequential organ failure assessment
SAPS II: Simplified acute physiology score II
ICU: Intensive care unit
PFF: Proximal femur fractures
SD: Standard deviation
OR: Odds ratio
